# Machine Learning Predicts Drug Metabolism and Bioaccumulation by Intestinal Microbiota

**DOI:** 10.3390/pharmaceutics13122001

**Published:** 2021-11-25

**Authors:** Laura E. McCoubrey, Stavriani Thomaidou, Moe Elbadawi, Simon Gaisford, Mine Orlu, Abdul W. Basit

**Affiliations:** Department of Pharmaceutics, UCL School of Pharmacy, University College London, London WC1N 1AX, UK; laura.mccoubrey.18@ucl.ac.uk (L.E.M.); stavriani.thomaidou.20@ucl.ac.uk (S.T.); m.elbadawi@ucl.ac.uk (M.E.); s.gaisford@ucl.ac.uk (S.G.); m.orlu@ucl.ac.uk (M.O.)

**Keywords:** artificial intelligence, classification, semi-supervised learning, gastrointestinal microbiome, drug stability, drug discovery and development, pharmacokinetics, in silico prediction, principal component analysis, feature selection

## Abstract

Over 150 drugs are currently recognised as being susceptible to metabolism or bioaccumulation (together described as depletion) by gastrointestinal microorganisms; however, the true number is likely higher. Microbial drug depletion is often variable between and within individuals, depending on their unique composition of gut microbiota. Such variability can lead to significant differences in pharmacokinetics, which may be associated with dosing difficulties and lack of medication response. In this study, literature mining and unsupervised learning were used to curate a dataset of 455 drug–microbiota interactions. From this, 11 supervised learning models were developed that could predict drugs’ susceptibility to depletion by gut microbiota. The best model, a tuned extremely randomised trees classifier, achieved performance metrics of AUROC: 75.1% ± 6.8; weighted recall: 79.2% ± 3.9; balanced accuracy: 69.0% ± 4.6; and weighted precision: 80.2% ± 3.7 when validated on 91 drugs. This machine learning model is the first of its kind and provides a rapid, reliable, and resource-friendly tool for researchers and industry professionals to screen drugs for susceptibility to depletion by gut microbiota. The recognition of drug–microbiome interactions can support successful drug development and promote better formulations and dosage regimens for patients.

## 1. Introduction

Over 150 drugs are recognised as being susceptible to metabolism or bioaccumulation by intestinal microbiota [1,2,3,4,5,6,7,8,9,10,11,12,13,14,15]. These direct effects on drug concentration can lead to significant inter-individual variability in pharmacokinetics, arising due to differences between individuals’ gut microbiome compositions [16,17,18]. Microbial drug metabolism or bioaccumulation (termed henceforth as depletion) is often dependent upon the production of specific enzymes that may be variably expressed between patients [3,19,20,21,22,23]. For example, digoxin is inactivated by strains of *E. lenta* that produce the cardiac glycoside reductase (CGR) enzyme [24]. Research has shown that the abundance of the CGR gene relative to *E. lenta* concentration in patients’ faeces is significantly correlated with their ex vivo metabolism of digoxin, providing strong evidence that patients colonised by CGR-encoding bacteria metabolise digoxin to a greater extent in vivo [25]. Another drug whose microbial metabolism has been explored, with consideration for inter-individual variability, is tacrolimus [26]. Lee et al. have highlighted that the abundance of *F. prausnitzii* in patients’ stools is positively correlated with their tacrolimus dosing requirements [27]. This relationship is thought to arise because *F. prausnitzii* transforms tacrolimus to a metabolite known as M1, which has 15-fold lower immunosuppressant activity than the original drug [26].

The inter-individual pharmacokinetic variability arising from the microbial depletion of drugs could contribute towards treatment failure or toxicity in some patients, in addition to difficulties finding an optimum dose [28,29,30]. Further, pharmacokinetic variability could risk the progression of novel treatments through clinical trials to market approval. Despite these risks, the susceptibility of drugs to microbiota depletion is not routinely tested during preclinical or clinical development [31]. In rare cases when microbial metabolism is explored, it is usually conducted to determine drugs’ stability in the colonic environment rather than to study pharmacokinetic variability [7]. Due to its underexplored nature, there is currently no universally accepted method for the confirmation of microbial drug bioaccumulation [3]. Common methods used to quantify microbial drug metabolism involve incubating drugs in either human or animal faecal slurries, microbial cultures, and less commonly in intestinal fluids to measure drug degradation over a defined period (usually ≤24 h) [1,2,6]. Experimental determination of drugs’ susceptibility to microbial depletion can be a time-consuming and resource-intensive process. High-throughput screening of many investigative active pharmaceutical ingredients (APIs) may not be feasible in industry for several reasons. Firstly, drug development pipelines may not have the necessary time required to measure many drugs’ degradation and metabolite formations, as drug-specific high-performance liquid chromatography or mass spectrometry methods are frequently required [2]. Moreover, it is desirable to minimise the use of animals, and sourcing either human faecal or intestinal fluid samples can be difficult and expensive. For this reason, in silico methods of predicting drugs’ microbiome depletion hold significant potential [32,33,34,35,36].

To date, there are a few examples of the in silico prediction of microbial drug depletion [37]. In 2017, Sharma et al. used random forest ML to develop a predictive model known as DrugBug [38]. The group identified 324,697 metabolic enzymes from 491 gut bacterial genomes. Non-drug substrates of these bacterial enzymes (*n* = 1609) were then retrieved from the KEGG database (a resource containing thousands of biochemical interactions). The substrates were used as a training dataset to classify the bacterial enzymes most likely to metabolise drugs. Though the model achieved >90% accuracy in predicting the non-drug substrates’ associated enzymes, the field has evolved since, requiring new input considerations. Firstly, the model was developed at a time when only a handful of drugs had characterised bacterial metabolic reactions, resulting in an outdated model. Further, the outcome of drug stability was not considered (the model assumes all drugs were metabolised by bacterial enzymes); however, it is now suspected that the majority of drugs are resistant to microbial transformation [1,2]. In 2019, as part of their high-throughput screening study, Zimmerman et al. used a hierarchical clustering algorithm to examine the functional groups that increase drugs’ risk of being microbially depleted [1]. They observed that drugs containing urea, azo, lactone, and nitro functional groups were more likely to be depleted by at least one of the tested 76 gut bacterial strains. Whilst this explorative analysis is interesting, it should be explained with a quantifiable means to assess untested drugs’ risk of microbial depletion to provide further insight into microbiome activity. Elsewhere, Elmassry et al. evaluated drugs’ risk of metabolism by bacterial β-glucuronidases using a common substructure algorithm [39]. Drugs inferred to undergo reaction with microbial β-glucuronidases, due to their known reaction with hepatic β-glucuronidases, were clustered based on their chemical structures. Structural similarity of new drugs with those included in the study could facilitate predictions for untested β-glucuronidase metabolism. However, such predictions would be based on the untested assumption that drugs in the training set really do undergo metabolism by microbial β-glucuronidases.

This study aimed to develop a classification algorithm capable of predicting whether small molecule drugs are susceptible to direct depletion (i.e., metabolism or bioaccumulation) by gut microbiota. The model output is binary, i.e., depleted or not depleted, and includes a level of predictive confidence. This technology could become a valuable tool for in silico prediction of drug–microbiota interactions, and the compounding effects on bioavailability and pharmacokinetic variability.

## 2. Materials and Methods

### 2.1. Dataset Curation and Labelling

**Figure 1:**
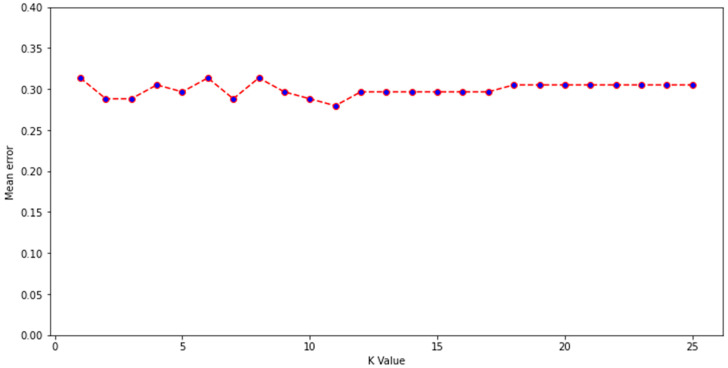
Mean error for K values of 1–25 during construction of a K-nearest neighbour algorithm developed to label unlabelled drugs.

Experimental data describing the depletion of drugs by gut microbiota were compiled from several studies, with the majority from work by Zimmerman et al. and Javdan et al. [1,2,3,6,7,10,12]. Zimmerman et al. incubated 271 drugs independently with 76 gut bacterial isolates anaerobically for 24 h. Drugs were labelled as being significantly (*p* < 0.05) depleted by at least one bacterial strain if ≥20% reduction in starting concentration occurred [1]. Javdan et al. anaerobically incubated 438 drugs in the presence of gut microbiota sourced from a single human donor (sex not disclosed) [2]. Drugs were labelled as metabolised if the drug was observed to be entirely consumed and a new metabolite was formed after 24 h, in at least 2 of 3 independent experiments. Drugs from other included studies were incubated under anaerobic conditions for 24 h in faecal slurry sourced from multiple healthy human donors (both sexes) and labelled as metabolised if they were significantly degraded compared to controls [6,7,10,12]. In this manner, drugs were labelled into two categories: depleted or not depleted. Where the same drug was tested in two studies (this included multiple salt forms of the same drug), and results disagreed, another study from the literature was sought to provide a 3rd opinion and enable the assignment of a label. Importantly, only studies based on gut bacterial isolates or human faecal/intestinal fluid were considered; studies examining drug metabolism in animals were not included, as the microbiome composition of animals is significantly different to that of humans [40]. Where a 3rd study could not be found, then drugs were placed into a temporary category known as undefined. These drugs (*n* = 86) were then assigned a label using a K-nearest neighbours algorithm trained on the labelled drugs (*n* = 469). Model inputs were the 86 unlabelled drugs attached to 200 physicochemical parameters from Python’s RDkit library (version 2021.03.1) combined with Morgan fingerprints (radius 2, 1024 bits). K = 11 was chosen for the K-nearest neighbours algorithm, as this value was found to have the lowest mean error rate during training, from K values of 1–25 (Figure 1). A mean error rate of 0 signifies that no errors were made by the model during classification of the training dataset, whereas mean errors closer to 1 demonstrate higher instances of incorrect prediction. A k value of 11 indicates that a sample was assigned a group based on the classifications of its nearest 11 neighbours in the multidimensional feature space. Following this, a labelled dataset of 555 drugs was formed. Because the dataset was unbalanced (drugs labelled as not depleted = 411; drugs labelled as depleted = 144), which can affect the reliability of machine learning models, 100 drugs from the not depleted category were removed from consideration using a random seed of 10 in Python. This resulted in 455 drugs being considered in a more balanced dataset (311 not depleted; 144 depleted). The predictive performance of the original and more balanced datasets was assessed.

### 2.2. Feature Generation

Simplified molecular-input line-entry system (SMILES) notations were obtained from PubChem for each drug. Based on these SMILES structures, two types of molecular fingerprints were assigned to each drug: Morgan fingerprints (radius 2, 1024 bits) and 200 physicochemical parameters from Python’s RDkit (version 2021.03.1). Morgan fingerprints are one of the most popular chemical descriptors used for small molecules and provide a fingerprint based on multiple substructures around each atom in a molecule [41,42,43]. In comparison, the descriptors generated from RDkit include more functional, property-based features of drugs such as molecular weight and SLogP. Both Morgan and physicochemical fingerprints were investigated as drugs’ inputs for the machine learning models, trialled together and in isolation to determine the best descriptors.

### 2.3. Data Preprocessing and Visualisation

Drug labels were encoded as 0 (not depleted) or 1 (depleted) using Sklearn’s LabelEncoder. To remove noise arising from unit variance, drug features were standardised using the StandardScaler tool in the Python sklearn.preprocessing library. The dataset was also checked for NA values, and none were present. To visualise the spread of data, a principal component analysis (PCA) algorithm was applied using the standard PCA tool in Python’s Sklearn package (random state = 0). The entire labelled dataset was decomposed into principal components, and the percentage explained variances of the top 10 components were elucidated using a Scree plot. Following this, the top 2 principal components were plotted with drugs labelled as depleted/not depleted.

### 2.4. Development of Machine Learning Models 

Eleven ML techniques were investigated in this study to capture performance across a range of learning styles: extra trees, random forest, K-nearest neighbours (kNN), multilayer perceptron (MLP), decision tree, support vector machine (SVM), gradient boosting, logistic regression, stochastic gradient descent (SGD), perceptron, and passive aggressive classification. A background on various ML methodologies has been published by Badillo et al. [44]. During initial development, all models were used in their baseline state, imported from Python’s Sklearn library (random state = 0 where applicable). The 11 baseline models were assessed for their ability to predict drugs’ microbiome status using 4 performance metrics with Sklearn: mean area under the curve of the receiver operating characteristic (AUROC), weighted precision, weighted recall, and balanced accuracy. Weighted and balanced metrics were used to offset bias arising from the imbalance between the number of depleted vs. not depleted drugs in the dataset. Weighting performance involved calculating metrics for both classes and then finding their average based on the number of instances in that class. Performance metrics were chosen to give a global understanding of models’ performances [45]. The AUROC score summarises a model’s true positive (TP) prediction rate as a function of false positive (FP) prediction rate. Precision equals the number of TPs divided by the total number of positives (TPs + FPs) generated by a model, and as such it captures the likelihood of positive cases being overestimated (type 2 error). Recall is defined as the number of TPs divided by the number of actual positives (TPs + false negatives (FNs)) within a model, thus measuring the risk of positive cases being missed (type 1 error). Finally, balanced accuracy equals the number of correct predictions, weighted by the number of true and false samples in the dataset, divided by 2 (Equation (1)). Each model’s performance metrics were calculated using the ShuffleSplit cross-validation tool in Sklearn (number of splits = 10, test size = 20%, random state = 0). Using this form of cross-validation, the entire dataset was shuffled and split into 10 random groups, within which 80% of data were used for training and the remaining 20% for testing. Using cross-validation leads to higher trust in models’ performance, as scores are generated using several different test datasets, thus also providing protection against model overfitting. Here, models’ performance scores are presented as averages (means) and standard deviations across all cross-validation folds.

(1) $$\mathrm{Balanced}\;\mathrm{accuracy}=\frac{1}{2}\left(\frac{\mathrm{TP}}{\mathrm{TP}+\mathrm{FN}}+\frac{\mathrm{TN}}{\mathrm{TN}+\mathrm{FP}}\right)$$

### 2.5. Selection and Optimisation of Best Model 

The best ML model was selected by considering global performance. Where a model achieved the 1st, 2nd, or 3rd best score for a single metric, it was assigned 3, 2, or 1 point(s), respectively. Models’ scores across the 4 metrics were totalled and the models were ranked, with the best attaining the highest overall score. The best model was then optimised by hyperparameter tuning, using the RandomizedSearchCV function within Python’s Sklearn package (param_distributions = random_grid, n_iter = 50, cv = 3, verbose = 2, random_state = 0, n_jobs = −1). The parameters included in the randomised search were n_estimators, max_features, max_depth, min_samples_split, min_samples_leaf, bootstrap, and class_weight. The Python code for the final model is available in the Supplementary Materials, with which users can make predictions for untested APIs. With the model prediction (depleted/not depleted), there is a level of predictive confidence supplied by training an ActiveLearner (modAL active learning framework for Python version 0.4.1 (Szeged, Hungary)) based on the final model. This feature outputs classifier uncertainty (from 0.00–1.00) for each prediction, whereby lower uncertainty scores signify that the model has higher confidence in a specific prediction.

### 2.6. Data Analysis and Statistics 

A PC (running on operative system: Windows 10 64-bit, processor: Intel^®^ Core i7 3770 K (Santa Clara, CA, USA) (overclocked 4.5 GHz), RAM: 16 GB DDR3, and graphics card: Asus Phoenix GTX 1660 OC Edition (Taipei, Taiwan)) was used for data analysis and ML model construction. The ML dataset was compiled within Microsoft^®^ Excel^®^ for Microsoft 365 MSO (16.0.13231.20372) 64-bit. Dataset cleaning and preprocessing, and model construction and evaluation, were completed using Python version 3.9.0 (Wilmington, DE, USA) on Jupyter Notebook version 6.0.3 (San Francisco, CA, USA). All ML techniques were developed using Python’s scikit-learn package, version 0.23.2. Metrics used to assess models’ performance: AUROC, weighted precision, weighted recall, and balanced accuracy. The statistical difference between models was calculated using either a t-test (when comparing two models) or one-way ANOVA (when comparing > 2 models) using the Scipy package (version 1.7.1) in Python, with *p* < 0.05 taken as significant. The time taken to fit models (*n* = 3 for each measurement) was computed using Python’s time function. Feature shuffling was performed by randomly relocating features’ positions in the training dataset, followed by analysis of the ML model performance, to ensure the validity of the final model’s performance scores. The 3 shuffled datasets are available in the Supplementary Materials. Plots were constructed using the Matplotlib package in Python and OriginPro (version 2021b).

## 3. Results and Discussion

### 3.1. Unsupervised Learning

**Figure 2:**
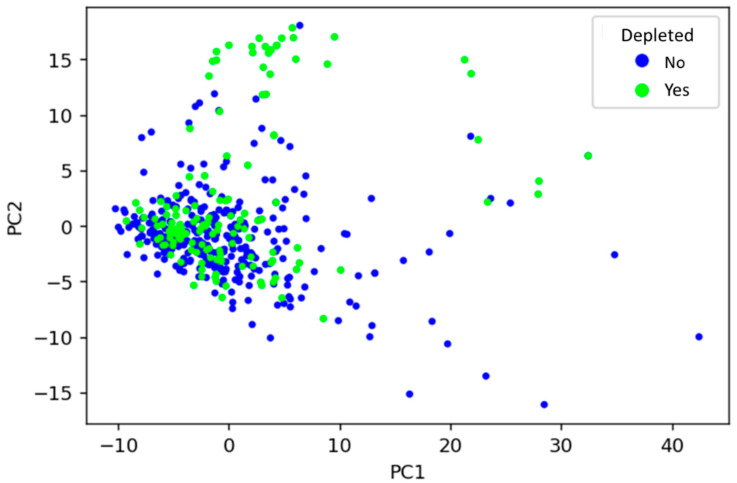
Visualisation of the relationship between drugs’ physicochemical features and whether they are depleted by gut microbiota, using principal component analysis. PC1: principal component 1, PC2: principal component 2.

To visualise how drugs’ physicochemical features relate to their microbial depletion status, the dataset was decomposed using PCA into principal components (PCs), and the first two PCs (accounting for around 6.5% of total variance) were plotted (Figure 2). PCA is an unsupervised learning technique that can find inherent differences in data without the need for a labelled dataset. PCA revealed that depleted drugs have similar physicochemical properties to those not depleted, depicted by many drugs sharing a similar physicochemical space regardless of label. That said, certain physicochemical fingerprints may be predictive of microbial depletion, as suggested by regions of mainly non-depleted drugs in the PCA plot. Alone, the PCA analysis cannot provide sufficient predictive power because distinct clusters of non-depleted/depleted drugs are not apparent in Figure 2. Therefore, numerous supervised ML techniques were subsequently explored to facilitate such predictions. 

### 3.2. Dataset Balancing

**Figure 3:**
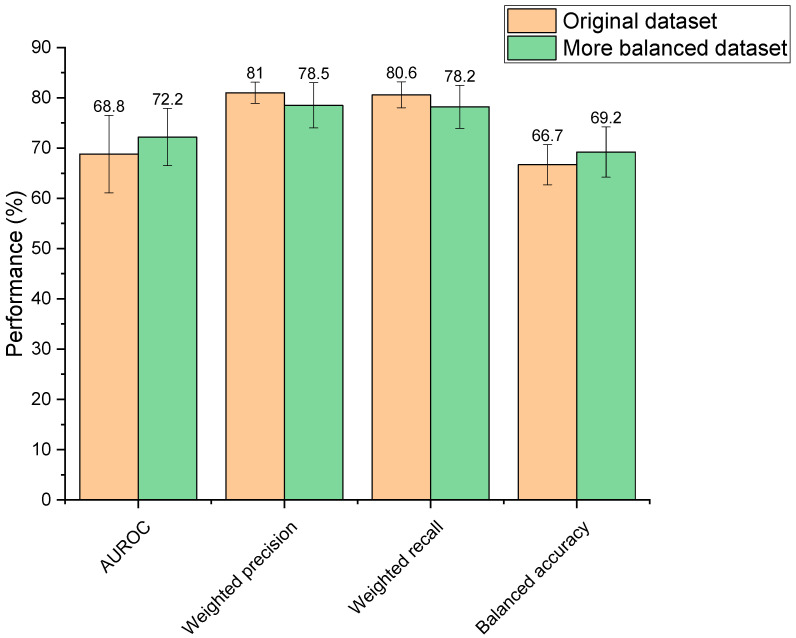
Performance of two extra trees machine learning models in predicting drugs’ microbial depletion status. Orange bars indicate scores for the model trained on the original unbalanced dataset. Green bars indicate scores for the model trained on a more balanced dataset.

Increasing the balance between the two classes within the training dataset (depleted vs. not depleted) resulted in performance changes; however, these were not statistically different (AUROC: *p* = 0.31, weighted precision: *p* = 0.16, weighted recall: *p* = 0.14, balanced accuracy: *p* = 0.26) (Figure 3). Though observed changes were insignificant, it is generally recognised in the field that unbalanced datasets need amendment to produce a reliable ML model, otherwise classifications may be biased towards the overrepresented group [44]. Moreover, the more balanced dataset was significantly faster to train than the unbalanced set (2.63 s compared to 3.07 s, *p* = 3.44 × 10^−7^) due to its smaller size. Based on this reasoning, the more balanced dataset was selected for use going forward.

### 3.3. Feature Selection 

**Figure 4:**
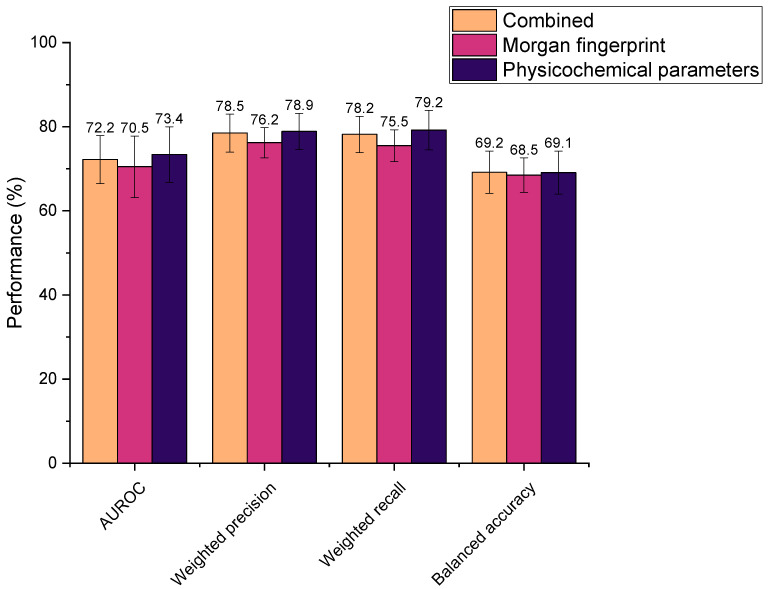
Performance of extra trees machine learning models in predicting drugs’ microbial depletion status. Bar colours indicate the features used to describe drugs in the training dataset. Combined: both Morgan fingerprints (radius 2, 1024 bits) and 200 physicochemical parameters (from Python’s RDkit).

Three different feature selections were explored in this study, where all were found to yield high metric scores (Figure 4). Performances were determined as statistically indifferent across the sets (AUROC: *p* = 0.66, weighted precision: *p* = 0.19, weighted recall: *p* = 0.34, balanced accuracy: *p* = 0.95). Therefore, all could be feasibly used as drug features in the ML models. However, the physicochemical set has fewer features than the Morgan fingerprints (by 824 features), thus it was hypothesised that ML model training using physicochemical parameters alone would be computationally less intensive than when incorporating Morgan fingerprints. This was reflected in the time taken to train models, whereby the combined feature set and Morgan fingerprints alone required an average of 2.63 s and 2.67 s to train, respectively, compared to just 1.71 s for the physicochemical parameters in isolation. Accordingly, the physicochemical feature set needed significantly less time to train the representative extremely randomised trees (extra trees) ML model than when combined with Morgan fingerprints or the Morgan fingerprints alone (*p* = 6.85 × 10^−8^ and 7.53 × 10^−8^, respectively). Computational efficiency is an important consideration when developing ML models, especially in the context of this study, in which numerous models were screened. In practice, more efficient models would be better suited to the high-throughput in silico screening of drug–microbiota interactions, as more predictions could be generated in a given timeframe. In addition, utilising the smaller physicochemical feature set ensured that the number of features was comparable to the number of drug–microbiota observations, minimising overfitting [46,47]. In recognition of these factors, all subsequent models were trained on drugs’ physicochemical features alone.

### 3.4. Supervised Machine Learning

#### 3.4.1. Baseline Models

**Figure 5:**
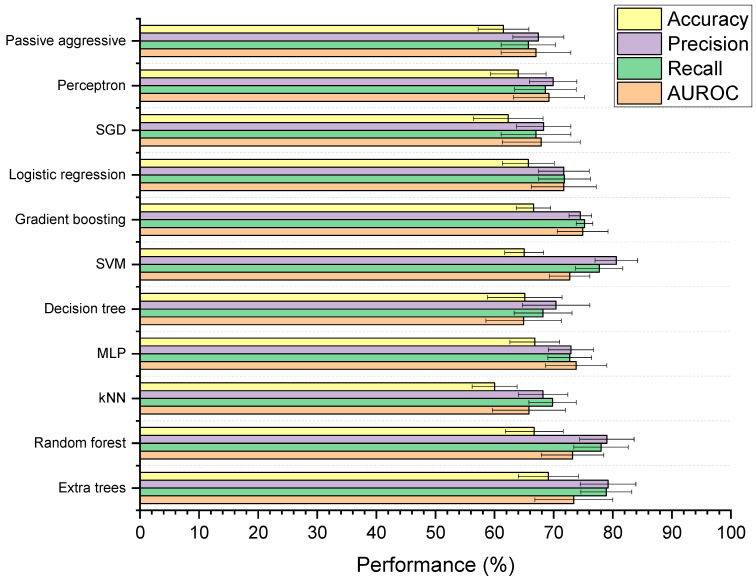
Performance of machine learning (ML) models designed to classify drugs as either susceptible or not susceptible to depletion by gut microbiota. Accuracy is balanced, and precision and recall are weighted. The 11 ML techniques were: extra trees, random forest, K-nearest neighbours (kNN), multilayer perceptron (MLP), decision tree, support vector machine (SVM), gradient boosting, logistic regression, stochastic gradient descent (SGD), perceptron, and passive aggressive classification.

Figure 5 shows the performances of the 11 supervised ML models developed to classify whether drugs are microbially depleted or not. Based on mean scores alone, the extra trees model received the highest ranking (with nine ranking points) followed MLP, SVM, and random forest (holding four ranking points each). However, when examined for significant differences, the models were found to be statistically similar based on AUROC (*p* = 0.98) and balanced accuracy (*p* = 0.28). For weighted precision and recall, the MLP model had significantly lower performance than the extra trees, SVM, and random forest models (*p* < 0.05), though extra trees, SVM, and random forest were all statistically indiscriminate. The extra trees model was selected for further optimisation, as it is a reliable tree-based method that can learn both linear and non-linear relationships, and has been successfully used to model drug–microbiome interactions in the past [48]. Compared to a simple decision tree, extra trees has the benefit of utilising multiple randomised trees to generate predictions on subsections of data, where final predictions are averages across all trees [49]. This feature provides added control against overfitting and can improve predictive accuracy [50]. The extra trees model in this study had significantly higher weighted precision (*p* = 0.002), weighted recall (*p* = 0.0001), and AUROC (*p* = 0.01) scores than the decision tree. Extra trees also has the added benefit of being more computationally efficient and reducing the variance of the trees compared to random forest, making it more generalisable to new data [51,52]. Here, the extra trees model was found to be more computationally efficient than the random forest model, requiring on average 0.49 s less to train (*p* = 5.84 × 10^−6^).

#### 3.4.2. Hyperparameter Optimisation

**Figure 6:**
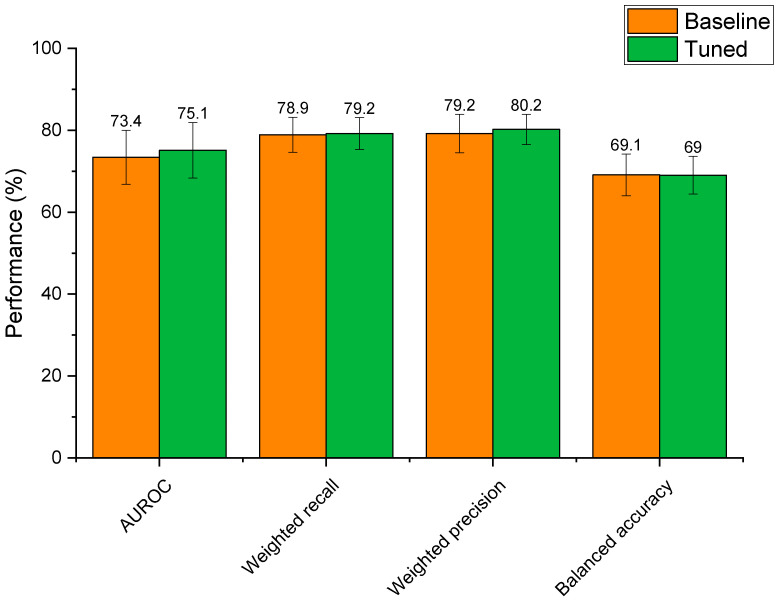
Performance of the baseline extra trees model compared to the extra trees model with hyperparameter tuning.

Optimisation of the extra trees model’s hyperparameters led to altered performance scores that were statistically indifferent to the baseline model (Figure 6). Whilst mean AUROC, weighted recall, and weighted precision increased post tuning, these differences were not significant (*p* > 0.59 for all metrics). This reflects that the baseline extra trees model was already well suited to modelling relationships between drug features and microbial depletion. That said, hyperparameter tuning reduced standard deviations for weighted recall, weighted precision, and balanced accuracy scores, demonstrating that the tuned model had slightly lower variability than the baseline model. In the case of AUROC, the standard deviation of the tuned model was only 0.2% lower than the baseline model. Based on this, the tuned extra trees algorithm was selected as the final model for the prediction of microbial drug depletion. The final model parameters were: n_estimators: 1400, min_samples_leaf: 1, max_features: ‘sqrt’, max_depth: 90, class_weight: ‘balanced’, bootstrap: False. Final metrics were calculated as: AUROC: 75.1% ± 6.8; weighted recall: 79.2% ± 3.9; weighted precision: 80.2% ± 3.7; and balanced accuracy: 69.0% ± 4.6. These metrics represent a model with high predictive performance, capable of predicting microbial drug depletion far better than human guess (50%) in a task that has never been accomplished before. The slightly lower balanced accuracy score may demonstrate that negative cases of drug depletion were more difficult to predict than positive cases, as the other three metrics focus mainly on positive prediction rates (see Section 2.4). The especially high precision score demonstrates that the model is very unlikely to make a type 2 error, in which a positive case of drug depletion is mistaken for a negative case. In practice, a low type 2 error rate is very important, as researchers can trust predictions that identify drugs as having a high risk of microbial depletion. This ensures that resources will not be wasted if only drugs predicted to be depleted are to be experimentally tested.

#### 3.4.3. Feature Shuffling

**Figure 7:**
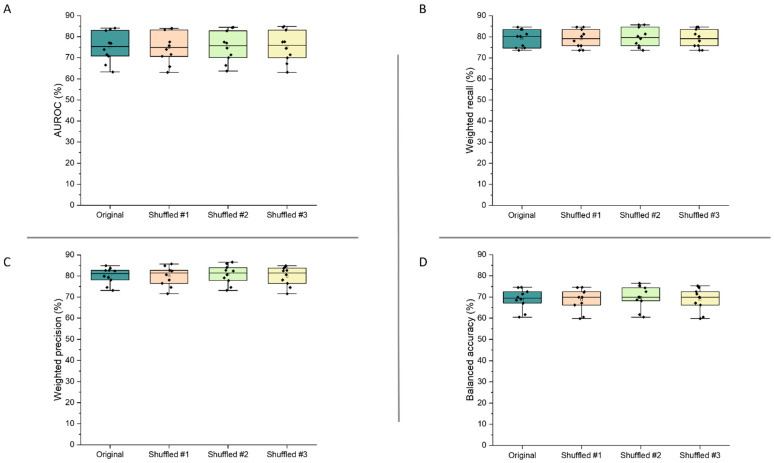
AUROC (**A**), weighted recall (**B**), weighted precision (**C**), and balanced accuracy (**D**) results from assessment of the final extra trees model by cross-validation, when feature order was shuffled in the training dataset (number of shuffles = 3).

Figure 7 demonstrates the validity of the final model, as the performance metrics were not significantly altered following shuffling of the features within the dataset (*p* > 0.95 for all metrics). These results show that the model’s high performance can be trusted, as slight changes to its inputs did not alter predictive performance.

In practice, a positive prediction for drug depletion can be taken to infer a ≥60% reduction in drug concentration during exposure to intestinal microbiota for 24 h, as this percentage sits at the intercept of the cut-off points used by Zimmerman et al. (≥20% reduction) and Javdan et al. (total reduction), whose data formed the majority of the ML model training dataset [1,2]. This information could be a useful tool in the pharmaceutical industry for the rapid screening of investigative molecules or the identification of unknown drug–microbiome interactions in research [48]. Work by Astra Zeneca has highlighted a strong correlation (R^2^ = 0.90) between drug stability in human faecal slurry and the fraction of drug absorbable in vivo in the colon [53]; therefore, predictions could also provide important information for the development of drugs with targeted release in the lower gastrointestinal tract. The pharmaceutical industry is rapidly embracing ML technology within their processes, and as the first of its kind, this model could offer significant value within the increasingly digitalised drug development pipeline [54,55,56,57,58,59]. In this model, microbial metabolism and bioaccumulation were combined within the same class because the data from Zimmerman et al. did not differentiate between the two mechanisms [1]. Drug accumulation by intestinal bacteria is a newly recognised concept, and as such sufficient data with which to predict metabolism and bioaccumulation as separate outcomes do not yet exist [3]. In the future, many more instances of bioaccumulation will likely be mapped, allowing distinction between metabolism and bioaccumulation.

## 4. Conclusions

In this study, a dataset describing 455 drugs’ depletion by intestinal microbiota was compiled by extracting data from the literature. Drugs were assigned into two classes (depleted/not depleted), with support from unsupervised learning, and after dataset balancing it was observed that providing physicochemical features as drug descriptors generated ML classifications with high average performance scores and good computational efficiency. The performances of 11 ML models with different learning techniques were compared and an extra trees model was selected for further optimisation, based on its high performance and efficient learning style. After hyperparameter tuning, the model achieved good performances (AUROC: 75.1% ± 6.8; weighted recall: 79.2% ± 3.9; weighted precision: 80.2% ± 3.7; and balanced accuracy: 69.0% ± 4.6) via cross-validation. The model maintained its performance in response to feature shuffling. This ML model is the first to accurately predict whether drugs will or will not be substantially metabolised or accumulated by intestinal microbiota. The algorithm could be a useful tool during the development of new drugs by allowing rapid screening of compounds’ susceptibility to microbial interactions that could significantly affect their pharmacokinetics. The code and dataset required to use the ML model can be found in the Supplementary Materials.

## Data Availability

The training dataset and Python code required to use the final machine learning model can be found in the Supplementary Materials.

## Supplementary Materials

The following are available online at https://www.mdpi.com/article/10.3390/pharmaceutics13122001/s1, Excel file S1 (training.xlsx): Training dataset for final extra trees model; Jupyter Notebook file S2 (Final code ML depletion (1).ipynb): Python code to use final extra trees model; Excel file S3 (training1.xlsx): Shuffled training dataset no. 1; Excel file S4 (training2.xlsx): Shuffled training dataset no. 2; Excel file S5 (training3.xlsx): Shuffled training dataset no. 3.
